# Topological organization of the “small-world” visual attention network in children with attention deficit/hyperactivity disorder (ADHD)

**DOI:** 10.3389/fnhum.2014.00162

**Published:** 2014-03-20

**Authors:** Shugao Xia, John J. Foxe, Ariane E. Sroubek, Craig Branch, Xiaobo Li

**Affiliations:** ^1^Gruss Magnetic Resonance Research Center, Albert Einstein College of Medicine, Yeshiva UniversityBronx, NY, USA; ^2^The Sheryl and Daniel R. Tishman Cognitive Neurophysiology Laboratory, Albert Einstein College of Medicine, Yeshiva UniversityBronx, NY, USA; ^3^Department of Pediatrics, Albert Einstein College of Medicine, Yeshiva UniversityBronx, NY, USA; ^4^Department of Neuroscience, Albert Einstein College of Medicine, Yeshiva UniversityBronx, NY, USA; ^5^Ferkauf Graduate School of Psychology, Yeshiva UniversityBronx, NY, USA; ^6^Department of Radiology, Albert Einstein College of Medicine, Yeshiva UniversityBronx, NY, USA; ^7^Department of Physiology and Biophysics, Albert Einstein College of Medicine, Yeshiva UniversityBronx, NY, USA; ^8^Department of Psychiatry and Behavioral Sciences, Albert Einstein College of Medicine, Yeshiva UniversityBronx, NY, USA

**Keywords:** ADHD, Attention, fMRI, graph theoretic techniques, functional connectivity, small world network, network-based statistic

## Abstract

**Background:** Attention-deficit/hyperactivity disorder (ADHD) is the most commonly diagnosed childhood psychiatric disorder. Disrupted sustained attention is one of the most significant behavioral impairments in this disorder. We mapped systems-level topological properties of the neural network responsible for sustained attention during a visual sustained task, on the premise that strong associations between anomalies in network features and clinical measures of ADHD would emerge.

**Methods:** Graph theoretic techniques (GTT) and bivariate network-based statistics (NBS) were applied to fMRI data from 22 children with ADHD combined-type and 22 age-matched neurotypicals, to evaluate the topological and nodal-pairing features in the functional brain networks. Correlation testing for relationships between network properties and clinical measures were then performed.

**Results:** The visual attention network showed significantly reduced local-efficiency and nodal-efficiency in frontal and occipital regions in ADHD. Measures of degree and between-centrality pointed to hyper-functioning in anterior cingulate cortex and hypo-functioning in orbito-frontal, middle-occipital, superior-temporal, supra-central, and supra-marginal gyri in ADHD. NBS demonstrated significantly reduced pair-wise connectivity in an inner-network, encompassing right parietal and temporal lobes and left occipital lobe, in the ADHD group.

**Conclusions:** These data suggest that atypical topological features of the visual attention network contribute to classic ADHD symptomatology, and may underlie the inattentiveness and hyperactivity/impulsivity that are characteristics of this syndrome.

## Introduction

Attention-deficit/hyperactivity disorder (ADHD) is the most commonly diagnosed psychiatric condition of childhood (Payne et al., 2011). It is a syndrome characterized by inattentiveness, impulsivity, and/or hyperactivity (American Psychiatric Association, 1994). Diagnosis and treatment of ADHD remain controversial due to the lack of firm understanding of its neurobiological causes (Halperin and Schulz, 2006).

Sergeant has proposed a cognitive-energetic model of ADHD incorporating three distinct levels: computational mechanisms of attention including four general stages (encoding, search, decision, and motor organization), state factors (such as effort, arousal, and activation), and an overriding management or executive system associated with planning, monitoring, detection of errors, and error correction(Sergeant, 2000, 2005). The cognitive-energetic model encompasses both top-down and bottom-up processes and draws attention to the fact that ADHD causes defects at all the three levels, which suggested that dysfunctional interplay between top-down and bottom-up information flow leads to impairment in the overall efficiency of information processing in ADHD (Sergeant, 2000, 2005). As such, this model implicates impaired systems-level topological organization of the functional brain networks responsible for sensory and cognitive information processing in ADHD.

The development of advanced network analysis methods such as graph theoretic techniques (GTT) and network-based statistics (NBS) has provided powerful approaches to characterization of the topological properties of functional brain networks from neurophysiological datasets (Watts and Strogatz, 1998; Bullmore and Sporns, 2009). These techniques have revealed that functional brain networks in the healthy human brain exhibit so-called “small-world properties,” which are typified by a high level of local clustering with short path lengths linking the working nodes within a given network. These features are believed to lead to a near optimal and highly economical organization for rapid synchronization and information transfer within a brain network by reducing wiring costs (Sporns and Honey, 2006; Bullmore and Sporns, 2009). One can reasonably ask why small-world properties are necessarily to be expected in neural networks. We know from multiple lines of evidence that the brain exhibits both local specialized processing units within relatively circumscribed regions or clusters of regions [e.g., area V4's role in color processing (Bartels and Zeki, 2000)] as well as distributed inter-regional interactions [e.g., the fronto-parietal attention control system (Dosenbach et al., 2008)]. That the brain operates in these two modes parallels nicely the topologies one finds in small-world networks, which comprise both high clustering and short path lengths. The former would support local specialized processing modules whereas the latter would support rapid integrative processing across distributed regions. In turn, the brain is space-delimited, residing as it does inside the skull, and while there are tens of billions of neurons in the human brain, there is a necessary space-dictated limitation upon the number of connections that can be made between processing units. Connections between distant regions are costly from both a space-occupying perspective as well as the energetics required to propagate signals along their greater extents and the greater volume of neural tissue that must be maintained to ensure their integrity [see (Kaiser, 2011) for an excellent discussion]. Here again, the reduced wiring costs that are characteristic of small-world topologies accord well with the wiring limitations that are clearly a feature of brain organization. It is of significant note that small-world properties are a feature of many self-organized and presumably well-optimized networks, including the internet, national power-supply grids and major transport systems (Bassett and Bullmore, 2006).

A number of recent studies have reported abnormal topological properties in the resting-state or default-mode networks (DMN) of children and adults with ADHD (Wang et al., 2009; Fair et al., 2010; Cocchi et al., 2012; Tomasi and Volkow, 2012). Compared to normal children, Wang and colleagues reported that ADHD children exhibited increased local efficiency of the whole brain network, significantly decreased nodal efficiencies in orbito-frontal, temporal, and occipital cortices and significantly increased nodal efficiency in the inferior frontal gyrus that are responsible for sensory-input, attention, and cognitive processing (Wang et al., 2009). Fair et al. found that correlated spontaneous activity of the brain regions within the DMN were reduced in children with ADHD (Fair et al., 2010). Children with ADHD also showed decreased connectivity in DMN and dorsal attention networks and enhanced connectivity within reward-motivation regions (striatum and anterior cingulate) (Tomasi and Volkow, 2012), and altered intrinsic connectivity in orbitofrontal-temporal-occipital and fronto-amygdala-occipital networks, detected during resting-state in young adult with ADHD (Cocchi et al., 2012). All of these previous findings suggest the presence of altered functional brain networks associated with attention and cognitive processing in ADHD. However, the topological features of functional brain networks expressly engaged during attention-demanding tasks have yet to be extensively investigated.

Tests of visual sustained attention performance, such as the continuous performance task (CPT), have been widely employed in the diagnosis of ADHD (Conners, 1995). During performance of a sustained attention task, as well as the obvious engagement of the front-parietal attention systems (Bressler et al., 2008), task performance also relies on diverse motor, sensory, and cognitive functions (Ballard, 1996). Thus, processing across multiple functional brain regions in a large scale network must be invoked and efficiently coordinated to successfully perform such a task. Here, we set out to apply the GTT and NBS techniques to fMRI data collected from children with ADHD and matched neurotypical controls as they performed a visual sustained attention task, to test the hypothesis that different topological and functional organizations of the visual attention network would be observed in ADHD. We also sought to analyze the relationships between the global and local network properties and clinical measures of the severity of the ADHD symptoms (DSM inattentiveness and hyperactivity-impulsivity scores), on the premise that atypical network organizations significantly contribute to the symptomatology and the neuropathology of ADHD.

## Methods

### Participants

A total of 48 right-handed children, aged from 9 to 15 years, were initially recruited to this study. Three participants were excluded from further analysis due to unacceptable levels of head motion during the scanning session, classified as deviations >1.0 mm in any of the six translation and rotation parameters or Mean Motion >0.25 mm in addition head motion assessment. One additional participant was excluded due to low response accuracy on the task during the fMRI recordings (<70%). This attrition left a total of 22 children with ADHD combined-type and 22 typically developing children (TDC) for inclusion in the main analyses. Diagnostic assessment of ADHD was performed using the revised long versions of Conners' Parent/Teacher Rating Scales (CPRS/CTRS) for both parent and teachers reports (Conners, 2008). The CPRS and CTRS reports provide information regarding the child's raw scores, how he or she compares to other children from a normative large sample—the T-scores. The diagnosis were confirmed with a parent interview using the Schedule for Affective Disorders and Schizophrenia for Children—Present and Lifetime Version (K-SADS-PL) (Kaufman et al., 1997). Inclusion criteria for the patient group met current DSM-IV criteria for combined-type ADHD. The TDC group included children who had T-scores <60 (<1 *SD*) on all Conners subscales. For both groups, we included the K-SADS-PL screening questions and supplements to rule out pervasive developmental disorders, substance use and abuse, and posttraumatic stress disorder. Similarly, oppositional defiant disorder with physical aggression (using DSM-IV diagnostic criteria), and all other current Axis I disorders (except for fear of the dark) were exclusionary. Children with any specific learning disorders were also excluded. The basic reading, mathematical reasoning, reading comprehension, and numerical operations subtests of the Wechsler Individual Achievement Test 2nd ed (WIAT-II) (Wechsler, 2001) were administered to determine the presence of impairments in reading or math. General exclusion criteria for both groups also included: chronic medical, neurological illness, or was taking systemic medication; specific or focal neurological disorder including epilepsy; treatment with any non-stimulant psychotropic within the past month; and contraindications for MRI scanning.

All subjects had normal or corrected-to-normal vision. Nine children in the ADHD group had been treated with short-effect stimulant medication (Ritalin). A 48 h wash-out period was undertaken by the care-givers of each of these patients before the day of MRI scanning to mitigate against potential medication effects on brain activations during fMRI task performance.

The children with combined-type ADHD were recruited from the Children's Evaluation and Rehabilitation Center at The Albert Einstein College of Medicine, and the Parnes Clinic at the Ferkauf Graduate School of Psychology. The TDC were recruited from local schools through newspaper advertisements, in collaboration with the Human Clinical Phenotyping Core of the Rose F. Kennedy Intellectual and Developmental Disabilities Research Center (RFK-IDDRC) at The Albert Einstein College of Medicine. This study received IRB approval for human subjects' research at Albert Einstein College of Medicine and all procedures consistent with the ethical standards laid out in the Declaration of Helsinki. After the study procedures were carefully explained, written informed consent was provided by all participants and their parents.

### Stimuli and task for fMRI

We employed a block-design CPT that consisted of 5 task blocks interleaved with 5 rest blocks. Each block lasted for 30 s. In each task block, a target sequence of 3 digits in red typeface (1–3–5, 2–4–6, 3–5–7, 4–6–8, or 5–7–9) was first shown in the center of the LCD screen, at the rate of one digit each 400 ms. Then, following a 1.8 s delay during which participants were required to remember the potential target sequence, a series of 9 additional 3-digitprobe sequences were presented sequentially in black typeface in a pseudo-random order, again at a rate of one digit every 400 ms. A 1.8 s response window followed each 3-digit probe sequence. In this period, participants were instructed to press the left button of a response pad using their index finger if the probe sequence matched the originally presented target sequence, and to use the middle finger to press the right button otherwise. During the rest blocks, a red cross was shown in the center of the LCD panel and participants were instructed to keep their eyes open, to maintain fixation, and to remain as relaxed and motionless as possible. The duration of the entire task was 5 min. Further details of design and rationale for the use of this CPT have been previously described (Li et al., 2012b).

### Data acquisition protocol

Imaging data were obtained using a 3.0 Tesla 32-Channel FreewaveAchieva MRI Scanner (Philips Medical Systems, Best, The Netherlands). fMRI data acquisition utilized whole brain gradient echo-planar imaging (EPI) sequence over a 230 ×128 acquisition matrix with 2 mm slice thickness, and 2 × 2 mm^2^ in-plane resolution, 41 slices, TE = 28 ms, TR = 2000 ms. High-resolution T1-weighted structural MRI data were also acquired for image registration (240 × 240 mm^2^ field of view (FOV) with 240 × 240 in-plane matrix and 1 mm partition thickness (1 mm isotropic resolution), TE = 4.6 ms, TR = 9.8 ms, α = 8o, SENSE factor = 2).

#### Data pre-processing

The fMRI data from each participant were pre-processed using the FSL/FEAT tools(Smith et al., 2004). Each imaging dataset was initially corrected for slice timing, spatial intensity normalization, and spatially smoothed with an 8 mm full-width and half maximum Gaussian kernel.

Head motion in fMRI data often causes position shifts of the brain structures, and can induce artifacts in the BOLD signals at each voxel that cannot be fully corrected. Severity of head motion is a key issue to be considered for inclusion criteria in any fMRI study. Recent studies utilizing resting-state fMRI data collected from large cohorts have demonstrated that patterns of head movement can significantly impact the dynamic patterns of resting-state BOLD signals. That is, motion is significantly associated with decreased functional connectivity between long-distance seed regions during the resting-state (Power et al., 2012), and is associated with increased short-distance local functional coupling, even after head motion spatial correction (Van Dijk et al., 2012). These studies suggest that head motion-induced artifact may be a critical component of resting-state BOLD signals, and may confound spontaneous brain dynamics. To test whether head motion-induced artifact adversely affected the visual attention task-based fMRI data collected in this study, traditional measurements of the six translation and rotation parameters were first calculated from the rigid body transformation for head realignment. In addition, head motion effects in the BOLD signals and their putative effects on functional connectivity patterns in our block-designed visual attention task-based fMRI data were assessed by using the frame-wise measurements of the six realignment parameters. The absolute values of the differentials of the time courses (Diff), the sum of the absolute values of the differentials of the six realignment parameters (FD) [calculation details provided in (Power et al., 2012)], and the Mean Motion [mean of the absolute values of the displacements along x-, y-, and z-axes compared to the previous volume (Van Dijk et al., 2012)] were in turn calculated from the whole fMRI data sets. Three spherical (*R* = 4 mm) regions of interest (ROIs) were chosen based on our previous findings which identified them as key nodes in the normal visual attention processing pathway (Li et al., 2012b). They were located in the right hemisphere thalamus [14, −26, 8], the right prefrontal lobe [45, 30, 4], and the left occipital lobe [−12, −94, 7]. Functional connectivities in the thalamo-prefrontal pairing and in the thalamo-occipital pairing were calculated from the time series of these three ROIs.

Finally, a high-pass temporal filter of 1/80 Hz was used to remove low-frequency noise. Non-brain structures were removed. The fMRI data were first aligned to the skull-stripedT1-weighted image from the same participant using an affine translation, and further registered to the MNI152 template using linear registration. Nine components including the six motion correlation parameters and nuisance signals (white matter, cerebrospinal fluid, and global signal) in the time series were regressed out from each fMRI dataset. The task-responsive activation maps in each individual and the average maps for each group were then generated by using the FSL/FEAT tool. The Z statistic image was thresholded using clusters determined by *Z* > 2.3 (a default setup of FSL/FEAT) and a cluster-corrected significance threshold of *p* < 0.05.

### Seed ROI detection and functional connectivity measures

A total of 68 spherical seed ROIs (radius = 5 mm) were identified from a combination (union) of the brain clusters that were significantly activated in the average activation maps in the ADHD or TDC groups. This combined activation map was parcellated according to the automated anatomical labeling (AAL) template (Tzourio-Mazoyer et al., 2002), which carved the cerebral cortex and subcortical structures into 90 anatomical areas bilaterally. Regions in cerebellum were not included for analysis at this stage, because we wanted to focus on the cortical and subcortical regions that are primary for visual attention processing circuits.

In this study, we interrogated the components of the fMRI signals in 0.015–0.125 Hz range, which has been demonstrated to contain the majority of the task-related hemodynamic information by multiple studies in block-design task-based fMRI data (Achard et al., 2006; Bassett et al., 2010; Ginestet and Simmons, 2011; Li et al., 2012a). Application of wavelet scales provided both noise suppression and permitted orthogonal assessment across multiple frequency bands of the hemodynamic response for each band, an approach that has been demonstrated to improve sensitivity of non-stationary signal analyses (Brammer, 1998; Bullmore et al., 2003; Ginestet and Simmons, 2011). Thus, we first tested three wavelet scales, to assess whether each band would have different sensitivities to the task design. Since our sampling frequency was 2 s (TR = 2 s) and wavelet kth scale provided information on the frequency band [2^−k−1^/TR, 2^−k^/TR], we examined the 0.015–0.031 Hz, 0.031–0.062 Hz, and 0.062–0.125 Hz bands, corresponding to wavelet scales 2, 3, 4, using the maximum-overlap discrete wavelet transform previously applied to time-frequency analyses of fMRI signals (Percival and Walden, 2000). As there were no significant sensitivity differences among the three sub-bands, we averaged the wavelet coefficients of the three frequency sub-bands for functional connectivity analysis. Pearson correlation of the average wavelet coefficients in each pair of the ROIs was calculated. The absolute values of the correlation coefficients were used to construct the functional connectivity matrix.

### Small-world network measures

The functional connectivity matrix was converted into a binary graph, by using the network cost as threshold. The cost *C_G_*, of a network (graph), *G*, was defined as following:

$$C_{G}=\frac{K}{N(N\text{-1})\text{/2}},$$

Where *N* and *K* are the total number of nodes (ROIs) and edges (functional correlations), respectively; *N*(*N*-1)/2 was the number of all the possible edges in the graph *G* (Latora and Marchiori, 2001). We investigated the network properties over a wide range of the cost values from 0.1 to 0.5 using increments of 0.01. According to existing studies, the selected threshold interval would allow the small-world properties to be properly estimated and the sub-networks to be connected with enough discriminatory power in functional connectivity (Achard and Bullmore, 2007). Then we calculated the two global metrics, global efficiency, *E_glob_*(*G*) and local efficiency, *E_loc_*(*G*), defined as:

$$E_{glob}(G)=\frac{1}{N(N-1)}\sum_{i\;\neq \;j\;\in \;G}\frac{1}{l_{ij}},\text{ and}E_{loc}(G)=\frac{1}{N}\sum_{i\;\in \;G}E_{glob}\left(G_{i}\right),$$

Where *l_ij_* is the shortest path length between nodes *i* and *j*; *E_glob_*(*G*) the global efficiency of the sub-network *G_i_* that is constructed by the set of nodes that are immediate neighbors of nodes *i* (Latora and Marchiori, 2001). The graph was considered to be a small-world network if it met the following criteria: *E_glob_*(*G_regular_*) < *E_glob_*(*G*) < *E_glob_*(*G_random_*) and *E_loc_*(*G_random_*) < *E_loc_*(*G*) < *E_loc_*(*G_regular_*), where *E_glob_*(*G_regular_*), *E_glob_*(*G_random_*), *E_loc_*(*G_regular_*), and *E_loc_*(*G_random_*) were the global and local efficiencies of node-and degree-matched regular and random networks.

We also investigated the nodal efficiency ${\text{E}}_{\text{nodal}}(\text{G},\text{ i})=\frac{1}{\text{N}-1}\sum_{\text{j }\in \text{ G}}{\frac{1}{\text{l}}}_{\text{ij}}$, which was a local measure to evaluate the communication efficiency between a node and all other nodes in the network *G* (Latora and Marchiori, 2001).

The network hubs were also identified in each group. In this study, a node was defined as a hub if the value of a measure, either degree (*D*) or betweenness-centrality (*BC*), of this node was significantly higher than the average value over all the nodes in the network. The degree (*D_i_*) of a node *i* was the number of edges connected to the node *i*. The betweenness-centrality (*BC_i_*) of node *i* was defined as the proportion of all the shortest paths between pairs of other nodes in the network that include node *i* (Sporns et al., 2007). A node with high *BC* is often interpreted as a gatekeeper that is able to control the information flow through that node (Langer et al., 2012). For each node, the hub measure (*D_i_* or *BC_i_*) was calculated at each cost value, from cost = 0.05 to 0.3, by using increments of 0.01. The mean value was then calculated for following statistical analysis (i.e., converted to z-scores using the distribution of averages for all nodes). We selected the mean of the measure between cost = 0.05 and 0.3, because this was the small-world regime identified in both groups. These standardized *z*-values were then tested with a normal distribution. A node *i* was defined as a network hub if the one side *p*-value in the *z*-test, *p* = 1 − Φ(*z_i_*), is less than 0.05 (i.e., the level of significance is α = 0.05), where Φ(·) was the standard normal cumulative distribution function.

### Bivariate NBS analysis

The NBS method, described in detail by (Zalesky et al., 2010), has been used to detect any pairwise associations that are significantly different between groups (Cocchi et al., 2012). In our study, the NITRC Tool, NBS v1.2 (http://www.nitrc.org/projects/nbs/), was applied to perform this part of analysis. The NBS seeks to identify any potentially connected regions formed by an appropriately chosen set of suprathreshold links. The topological extent of any such region is then used to determine its significance. It was performed as follows: (1) For each participant, the raw measurement-based functional connectivity matrix, constructed based on the (68 × 67)/2 = 2278 pairs of ROIs, was calculated. (2) A between-group *t*-test of the absolute Pearson correlation coefficient of each pair of the regions was calculated. This step was repeated independently in the 2278 pairs of ROIs. (3) Pairs of regions, which had a t statistic exceeding a threshold 3.5 [uncorrected *p* < 0.01, as recommended by NBS (Cocchi et al., 2012)], were selected as potential components for any interconnected networks that might have between-group difference. (4) The connected components from the resulting edges of step (3) were identified by using a breadth first search. (5) A family-wise error (FWE)-corrected *p*-value was then ascribed to each interconnected network by running the Permutation test. For each permutation, the subjects were randomly exchanged between the ADHD and the control groups. The NBS was then applied to the randomized data, and the size of the largest interconnected network was recorded. In our study, a total of 10,000 permutations were generated to yield an empirical null distribution for the size of the largest interconnected network. (6) As the final step, the corrected *p*-value for an interconnected network of size *k* was calculated as the proportion of the permutations for which the largest network was greater than or equal to *k*. As a result, we demonstrated the interconnected networks that had a FWE corrected *p* < 0.05.

### Group statistical analyses

Group comparisons of the participant characteristics were carried out using the chi-square test for sex, and unpaired two-sample *t*-tests for all other characteristics. A General Linear Model was applied to carry out group comparisons on the network measures (MathWorksInc, Natick, MA). Specifically, group differences in the global topological features, at each cost value from 0.05 to 0.5 using increments of 0.01, and the average nodal efficiency over the small-world regime at each node, were evaluated using an analysis of covariance, with age, IQ and sex as covariates. Multiple comparisons were corrected for the whole set of comparisons by using the False Discovery Rate (FDR) approach at α = 0.05. A linear regression analysis was utilized to assess possible relationships between the nodal efficiency measure of each node and the diagnostic measures of primary interest (the DSM inattentive, and hyperactive-impulsive scores) in the group of children with ADHD. Again, multiple comparisons were corrected for the nodes by using the FDR approach at α = 0.05. A significance threshold of *p* < 0.05 was applied for all tests.

## Results

As in Table 1, the ADHD and control groups did not differ significantly in demographic measures. All the subjects achieved >80% responding accuracy when performing the fMRI task. There were no significant between-group differences presented in the fMRI performance accuracy and reaction time data.

**Table 1:** **Demographic characteristics and diagnostic measures in both groups**.

**Measures**	**CON (*N*= 22)**	**ADHD (*N*= 22)**	**Statistic**	***p***
Age	12.1 ± 2.23	11.6 ± 2.86	*t* =2.35	0.07
Male/Female	10/12	12/10	χ^2^ = 3.50	0.71
Education(years)	6.2 ± 2.21	5.8 ± 2.81	*t* = 2.25	0.07
Mother's education	16.1 ± 3.46	14.7 ± 4.02	*t* = 2.09	0.08
Father's education	16.5 ± 3.55	14.8 ± 4.37	*t* = 1.77	0.06
IQ	114.7 ± 14.92	106.6 ± 16.21	*t* = 0.06	0.73
DSM-IV total T score	45.2 ± 5.33	74.7 ± 8.91	*t* = 4.67	<0.001
DSM-IV inattention T score	46.42 ± 6.61	75.3 ± 10.80	*t* = 4.91	<0.001
DSM-IV impulsivity T score	44.6 ± 4.75	70.6 ± 8.27	*t* = 4.44	<0.001

*CON, Neurotypical Controls; ADHD, participants with ADHD*.

**Figure 1:**
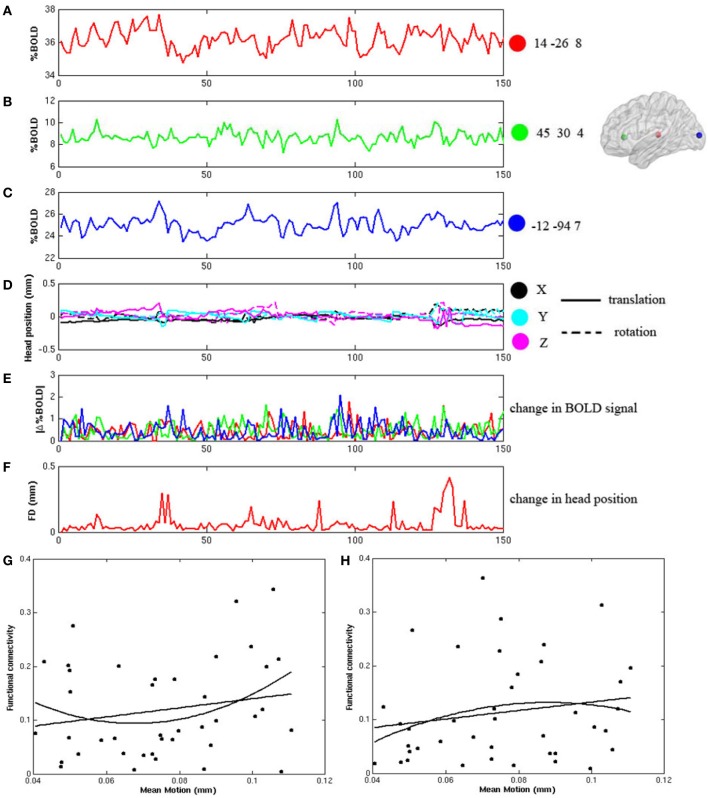
**Frame-wise changes in the fMRI signals during the block-design visual attention task are not related to the frame wise changes of head position after head motion correction. (A–C)** displays the fMRI time courses from 3 ROIs in a randomly selected participant. The ROIs are 8 mm diameter spheres centered on the coordinates of [14, −26, 8], [45, 30, 4], and [−12, −94, 7]; **(D)** shows the six frame-by-frame realignment parameters; **(E)** shows the absolute values of the differential of each time course in the three ROIs; **(F)** shows the sum of the absolute values of the differentials of the six realignment parameters (FD); **(G,H)** show that there are no significant correlations between Mean Motion and functional connectivity in either the thalamo-prefrontal pairing (linear: *r* = 0.20, *p* = 0.22; non-linear: *r* = 0.22, *p* = 0.17), or in the thalamo-occipital pairing (linear: *r* = 0.17, *p* = 0.28; non-linear: *r* = 0.16, *p* = 0.34).

During the frame-wise head motion analyses, we did not see any pattern of significant relationship between head motion and changes in the BOLD signals in any of the whole data sets. Figures 1A–F demonstrate the frame-wise head motion measures and comparisons with the BOLD signals and derivatives in a randomly selected subject. In addition, we did not find significant between-group differences in Mean Motion [ADHD 0.08 ± 0.031; Controls 0.07 ± 0.046; *p* = 0.7], and there were no significant correlations between Mean Motion and the functional connectivities in the thalamo-prefrontal and thalamo-occipital pairings across the whole sample (shown in Figures 1G,H).

**Figure 2:**
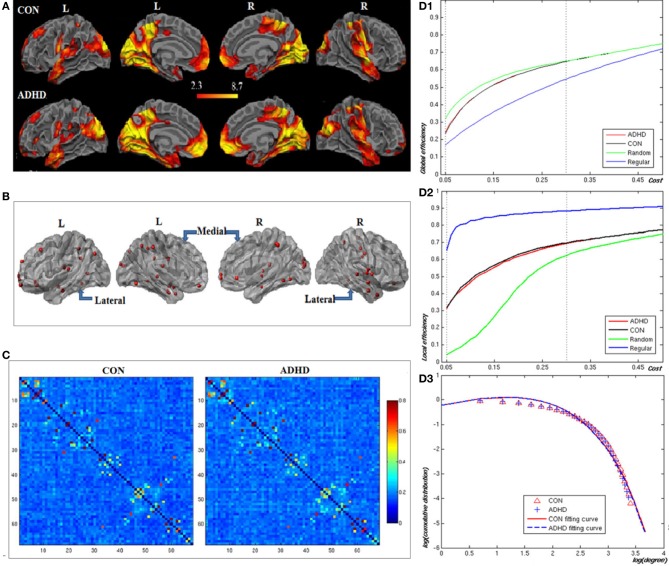
**Construction of the visual attention network in neurotypical controls (CON) and patients (ADHD)**. Panel **(A)** displays the voxel-based whole brain activation maps in both groups; Panel **(B)** the locations of the nodes in the network; Panel **(C)** the functional correlation matrices in both groups. Panels **(D1,D2)** display the global and local efficiencies respectively as a function of the cost of each network. Panel **(D3)** depicts the degree distributions of functional brain networks over the small-world regime. The degree distributions in both groups were fitted by an exponentially truncated power function of the form *P*(*k*) ~ *k*^α−1^e^−*k*/*k_c_*^.

Figures 2A,B, and Table 2 showed the locations of the 68 seed ROIs, which were selected from a combination (union) of the brain clusters that were significantly activated in the average activation maps derived from both groups. Figure 2C showed the functional correlation matrices in both groups. From Figures 2D1,D2, we observed that the locations of the global and local efficiency curves of both groups were between the corresponding curves of the random and regular graphs within the range 0.05 = cost = 0.3, known as a small-world regime (Achard and Bullmore, 2007). Compared to controls, the patients showed significantly decreased local efficiency over a range of network costs 0.18 = cost = 0.29. From Figure 2D3, the degree distributions in both groups were fitted by an exponentially truncated power of the form P(k) ~ k^α−1^e^−k/k_c_^ (normal controls: α = 1.622, k_c_= 5.127; ADHD: α = 1.631, k_c=5.062_). Stastical analysis indicated that there were no significant differences in the fitted parameters (α: t(36) = 1.306, *p* = 0.9; k_c_: t(36) = 0.659, *p* = 0.864 between two groups.

**Table 2:** **Node ROIs for functional brain network construction, which were identified from the activation maps in controls and patients, (L, left side; R, right side)**.

**Regions**	**Volume (mm^3^)^a^**	**Peak MNI coordinates**			***Z^b^***
		***x***	***y***	***z***	
L. Superior frontal gyrus (dorsal)	5736	−10	66	26	6.46
L. Superior frontal gyrus (medial)	9912	0	68	2	6.54
R. Superior frontal gyrus (medial)	3848	4	64	0	6.35
L. Orbitofrontal gyrus (superior)	416	−8	68	−8	5.7
L. Middle frontal gyrus	3120	−30	28	46	5.84
L. Orbitofrontal gyrus (middle)	440	−28	36	−14	5.95
L. Orbitofrontal gyrus (medial)	5320	0	68	−4	7.37
R. Orbitofrontal gyrus (medial)	5760	4	64	−6	7.73
L. Orbitofrontal gyrus (inferior)	1416	−28	34	−14	5.7
L. Rectus gyrus	1528	−4	52	−16	6.35
R. Rectus gyrus	1208	4	42	−16	6.64
L. Precentral gyrus	560	−56	−6	30	7.24
R. Precentral gyrus	6552	36	−20	44	7.68
L. Paracentral lobule	1072	−12	−34	50	5.55
R. Paracentral lobule	3832	12	−40	52	6.39
R. Supplementary motor area	1888	44	51	67	5.85
L. Postcentral gyrus	5232	−54	−8	26	7.34
R. Postcentral gyrus	13464	54	−12	36	7.68
L. Anterior cingulate gyrus	3016	−2	38	−8	6.63
R. Anterior cingulate gyrus	960	4	34	−6	6.01
L. Middle cingulate gyrus	5248	−8	−44	48	7.95
R. Middle cingulate gyrus	6344	8	−42	50	6.32
L. Posterior cingulate gyrus	2008	−8	−48	32	7.16
L. Superior parietal gyrus	1104	−16	−56	52	6.15
R. Superior parietal gyrus	368	16	−50	56	6.25
L. Inferior parietal gyrus	336	−36	−78	42	5.96
L. Precuneus	10472	−10	−44	48	8.15
R. Precuneus	9160	2	−50	52	7.88
L. Angular gyrus	3120	−40	−74	40	6.32
L. Supramarginal gyrus	1944	−64	−32	32	5.02
R. Supramarginal gyrus	6336	48	−16	30	6.44
L. Superior temporal gyrus	8656	−58	−2	−14	5.62
R. Superior temporal gyrus	14152	62	−8	2	7.13
L. Middle temporal gyrus	9608	−58	−4	−18	7.34
R. Middle temporal gyrus	5192	58	−6	−14	5.8
L. Inferior temporal gyrus	2544	−54	−4	−28	6.46
R. Inferior temporal gyrus	256	54	60	22	5.2
L. Temporal pole (superior)	1184	−54	6	−16	4.98
R. Temporal pole (superior)	2360	62	2	0	5.78
L. Temporal pole (middle)	1336	−58	−4	−18	4.84
R. Temporal pole (middle)	2312	28	12	−36	5.33
L. Heschl's gyrus	1288	−34	−24	14	4.56
R. Heschl's gyrus	1976	36	−26	16	6.83
L. Fusiform gyrus	8240	−28	−50	−12	7.56
R. Fusiform gyrus	7472	24	−64	−8	7.72
L. Superior occipital gyrus	7512	−16	−94	20	8.38
R. Superior occipital gyrus	5624	18	−90	26	7.32
L. Middle occipital gyrus	9472	−18	−90	18	7.11
R. Middle occipital gyrus	5152	26	−88	20	8.32
L. Lingual gyrus	13360	−20	−66	−12	7.56
R. Lingual gyrus	14136	24	−52	−10	8.01
L. Calcarine cortex	14440	−10	−60	6	7.96
R. Calcarine cortex	12144	8	−90	12	7.73
L. Cuneus	10704	−8	−74	18	7.93
R. Cuneus	9000	10	−92	16	7.52
L. Parahippocampal cortex	1784	−12	−34	50	5.55
R. Parahippocampal cortex	3832	12	−40	52	6.39
L. Insula	1432	−36	−16	16	5.43
R. Insula	3832	34	−14	6	6.39
L. Rolandic operculum	1912	−38	−16	18	5.47
R. Rolandic operculum	7008	66	−6	8	6.91
R. Putamen	1372	34	−14	2	6.16
R. Pallidum	248	28	−12	−2	5.37
L. Thalamus	504	−16	−26	0	5.01
R. Thalamus	416	16	−26	0	4.05
L. Amygdala	144	−20	−6	−18	3.64
L. Hippocampus	208	−22	−14	−24	5.25
R. Hippocampus	552	24	−12	−22	5.93

a *Is the number of activated voxels × 8 mm^3^; the ROIs were R = 5 spheres with origins located at the peak coordinates*.

b *is the z-value of the peak*.

Group comparisons of the nodal efficiencies of all the nodes over the small-world regime showed that patients had significantly reduced nodal efficiencies in the left superior orbito-frontal gyrus (*F* = 6.67, *df* = 1, 35, *p* = 0.014) and right superior occipital gyrus (*F* = 5.01, *df* = 1, 35, *p* = 0.032), but had significantly increased nodal efficiency in the left cuneus (*F* = 6.07, *df* = 1, 35, *p* = 0.019), after controlling for possible age, IQ and gender effects.

**Figure 3:**
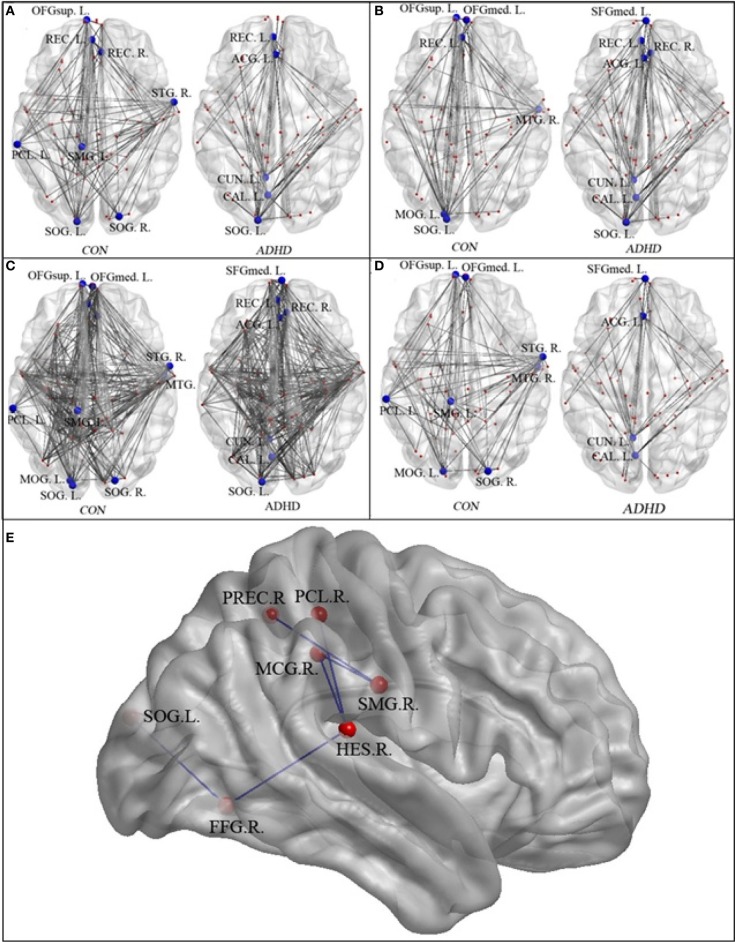
**The network hubs in the neurotypical controls (CON) and patients (ADHD)—regions are detailed in Table 2**. Panel **(A)** displays the hubs identified using the “between-centrality” measure for each group; Panel **(B)** the hubs identified using the “degree” measure; Panel **(C)** the combined hubs across both (i.e., the combination of “between-centrality” and “degree” measures); Panel **(D)** the between-group differences in the network hubs, displaying those nodes and edges (i.e., connections) that were unique to each group. Panel **(E)** provided the names and locations of the anatomical regions that constructed the inner-network over the entire visual attention network, which exhibited significantly lower pair-wise communications in the ADHD group when compared to the controls (SMG.R, right supra-marginal gyrus; PCL.R, right paracentral lobule; PREC.R, right precuneus; MCG.R, right middle cingulate gyrus; HES.R, right Heschl's gyrus; FFG.R, right fusiform gyrus; and SOG.L, left superior occipital gyrus).

Figures 3A–D and Table 3 detailed the anatomical regions and locations of the network hubs that were detected using average “degree” and “between-centrality” measures over the small-world regime in both groups. Group comparisons showed hyper-functioning network hubs in ACC of ADHD children, in contrast to hypo-functioning hubs in bilateral occipital lobes, the right temporal lobe and left paracentral and supramarginal gyri, in children with ADHD were compared to TDC.

**Table 3:** **Network hubs in the control group and ADHD group, (L, left side; R, right side)**.

**Network hubs**	**Degree**	**Between-centrality**
**CONTROLS**		
L. Orbitofrontal gyrus (superior)	✓	✓
L. Orbitofrontal gyrus (medial)	✓	
L. Paracentral lobule		✓
L. Rectus gyrus	✓	✓
R. Rectus gyrus		✓
L. Supramarginal gyrus		✓
R. Superior temporal gyrus		✓
R. Middle temporal gyrus	✓	
L. Superior occipital gyrus	✓	✓
L. Middle occipital gyrus	✓	
R. Superior occipital gyrus		✓
**ADHD**		
L. Superior frontal gyrus (medial)	✓	
L. Anterior cingulate gyrus	✓	✓
L. Rectus gyrus	✓	✓
R. Rectus gyrus	✓	
L. Calcarine cortex	✓	✓
L. Cuneus	✓	✓
L. Superior occipital gyrus	✓	✓

**Figure 4:**
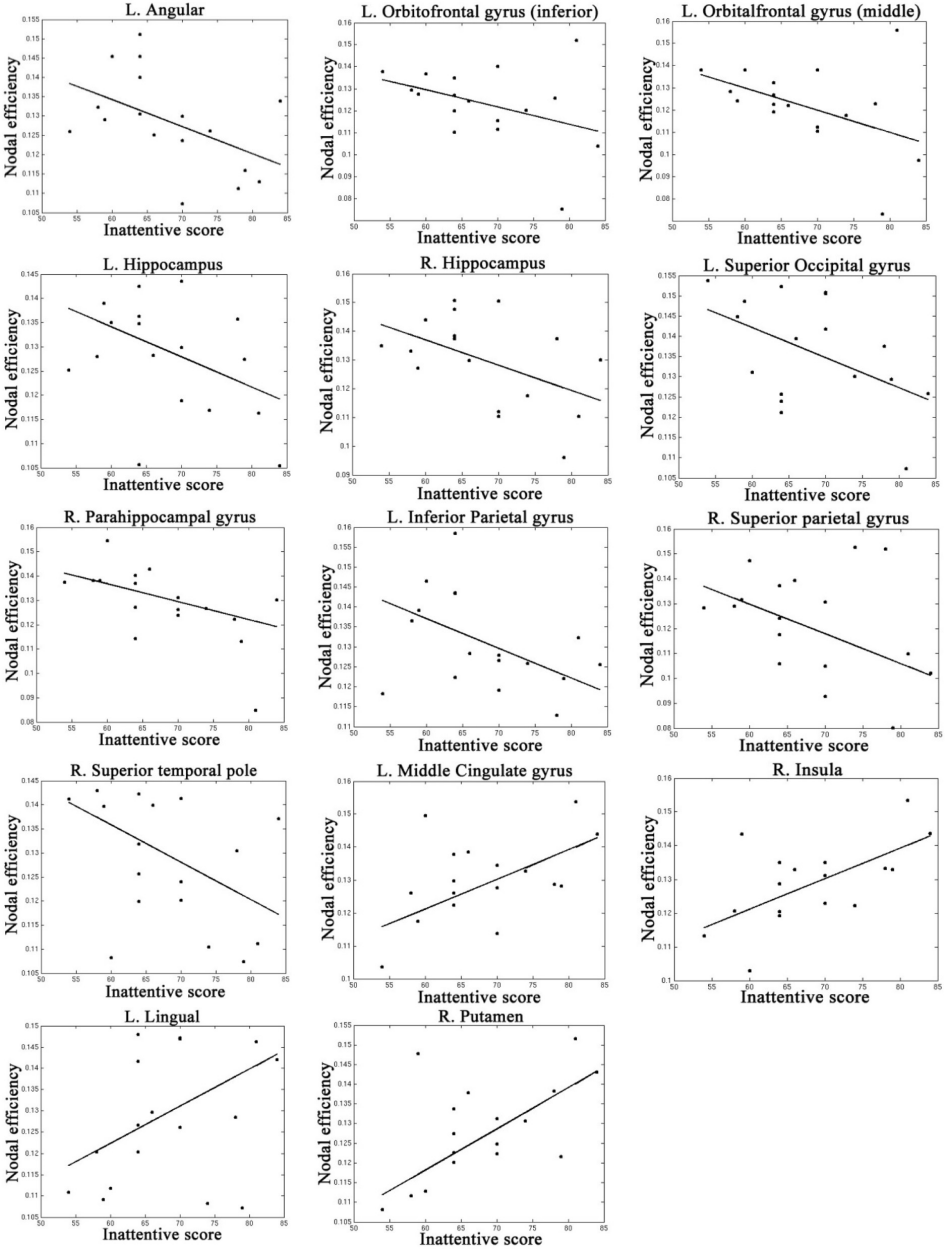
**Regions that showed significant correlations between the nodal efficiency and behavioral inattentiveness severity score**.

**Figure 5:**
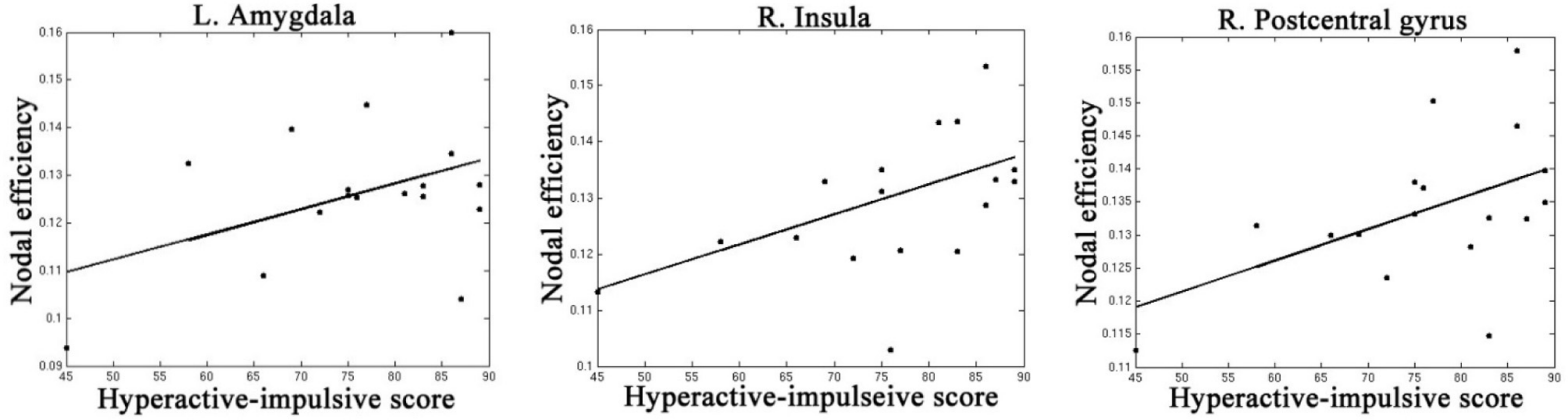
**Regions that showed significant correlations between the nodal efficiency and behavioral hyperactivity/impulsivity severity score**.

Table 4, Figures 4, 5 listed all the nodes where the nodal efficiencies were significantly correlated with diagnostic measures of ADHD (T scores of DSM-inattentive, DSM-hyperactive, and DSM-total symptoms). Nodal efficiency of the left supramarginal gyrus was negatively correlated with the inattentiveness scores; whereas that of the right supramarginal gyrus was positively correlated with the inattentiveness scores. We also observed that the nodal efficiencies of bilateral insular were positively correlated with the hyperactivity-impulsivity scores. The nodal efficiency of left superior temporal gyrus was negatively correlated with both of the diagnostic measures.

**Table 4:** **Brain regions that showed significant correlations between the nodal efficiencies and the clinical measures in ADHD participants, (L, left side; R, right side; r, the slope of least square linear estimation of the nodal efficiency vs. clinical measures)**.

**Regions**	**Statistics**		
	***r***	***F***	***p***
**SIGNIFICANTLY CORRELATE WITH INATTENTIVE SCORES**			
L. Angular gyrus	−0.0007	4.68	0.047
L. Orbitofrontal gyrus (inferior)	−0.0008	6.45	0.025
L. Orbitofrontal gyrus (middle)	−0.0010	12.73	0.003
L. Hippocampus	−0.0006	5.37	0.036
R. Hippocampus	−0.0009	4.68	0.047
L. Superior occipital gyrus	−0.0007	4.68	0.047
R. Parahippocampal gyrus	−0.0007	6.60	0.023
L. Inferior parietal gyrus	−0.0007	8.36	0.012
R. Superior parietal gyrus	−0.0012	4.82	0.045
R. Superior temporal pole	−0.0008	5.94	0.029
L. Middle cingulate gyrus	0.0009	10.69	0.005
R. Insula	0.0009	16.85	0.001
L. Lingual	0.0009	4.73	0.047
R. Putamen	0.0010	19.6	<0.001
**SIGNIFICANTLY CORRELATE WITH HYPERACTIVE-IMPULSIVE**			
**SCORES**			
L. Amygdala	0.0005	5.12	0.041
R. Insula	0.0005	8.49	0.011
R. Postcentral gyrus	0.0005	9.21	0.009

The NBS analyses found that compared to the controls, the ADHD group exhibited significantly reduced pair-wise connectivity in an inner-network, which consists of the right supra-marginal gyrus, right paracentral lobule, right precuneus, right middle cingulate gyrus, right Heschl's gyrus, right fusiform gyrus and left superior occipital gyrus (Figure 3E).

## Discussion

Findings here of altered topological features of the functional brain network driven by a visual sustained attention task in ADHD point to system-wide irregularities in regional interactions and global integration across the brain circuits. A key property of small-world networks is their robustness to minor nodal deletions and local communications failures, which allows these networks to continue functioning effectively under situations of injury or transient local disconnection (Kaiser et al., 2007). However, these networks can also have catastrophic failures when critical nodes are “attacked” or fail, for instance in the functional brain network driven by visual attention processing, when lesions to crucial nodes such as those in the prefrontal cortex or the right temporo-parietal junction occur (Kerkhoff, 2001). The findings of the GTT and NBS analyses in the present study suggest that such networks in ADHD contains fewer of these critical hubs, relying instead on greater involvement of a more delimited core of nodes (e.g., ACC).

We found that network hubs in the right temporal cortex, the left supramarginal, and paracentral gyri, which were observed in the networks of TDC, were essentially absent from the networks of the ADHD cohort (Table 3). A reasonable proposition is that when individuals are engaged in a protracted sustained attention task, there are likely to be relatively frequent transient attenuations or outright failures of activation within the key network hubs. In the case of TDC, these fluctuations of activation within the key nodal hubs may be relatively well tolerated because of the redundancy inherent in the system, since there are many such key hubs (Table 3). It follows, however, that reliance on a smaller core of critical network hubs in ADHD could lead to greater vulnerability to transient nodal failures, and ultimately to poorer performance on a given task. This thesis of increased network vulnerability will bear testing in future work. GTT measures of degree and between-centrality would allow for quantification of the number of key network hubs that are active at the individual participant level during task performance, which could then be assayed against the frequency of transient failures of attention. This would require the use of a more taxing task than the one used herein, since in the current study, we expressly employed a task in which all participants could achieve high performance rates.

In line with this notion of over-reliance on a more delimited set of key network hubs, the current data revealed significant hyper-functioning of the left ACC in ADHD (much higher between-centrality compared to that in controls), and significant positive correlation between nodal efficiency in the ACC and inattentiveness scores. The ACC has been implicated as a major coordinating hub within multiple cognitive control networks (Van Veen et al., 2001; O'Connell et al., 2007; Sheth et al., 2012), and the maintenance of appropriate arousal/vigilance states (Medford and Critchley, 2010). The fact that the left ACC was found to be substantially involved in task performance for ADHD children, but not for neurotypicals, is intriguing. One fairly straightforward inference is that over-reliance on a region that serves as a major coordinating center for multiple executive sub-functions could lead to increased susceptibility to transient failures as other processes (intrusions) access this hub. It is also telling that the ACC did not serve as a key hub in the attention networks of controls to solve this relatively basic CPT, suggesting perhaps, that controls engaged a network that may have been partially “sequestered” from critical multitasking circuitry. Our finding of aberrant ACC activation in ADHD is also consistent with a number of previous studies reporting functional abnormalities in this region during various tasks and at rest (Bush et al., 1999; Castellanos et al., 2008), as well as event-related potential studies showing decreased activation of error-awareness processes in the ACC of ADHD adults (O'Connell et al., 2009). These convergent approaches all point to the ACC as centrally involved in atypical regional communications of functional brain networks in ADHD.

Compared to the TDC, the ADHD group also showed significantly reduced nodal efficiencies in the left orbito-frontal gyrus. Previous work has suggested that the orbito-frontal gyrus plays a key role in modulating the connectivity between sensory and paralimbic brain regions and that it may mediate early top-down regulation of information processing(Rolls and Grabenhorst, 2008). As such, reduced nodal efficiency of this region may point to insufficiencies in top-down communications between executive control regions and visual processing regions. Existing imaging studies have also reported regional structural atrophy of this area in children with ADHD (Li et al., 2007), hypo-activation in this area in children with ADHD while they performed a visual attention task (Rubia et al., 2009), and reduced functional connectivity between this region with thalamus in children with ADHD, also during sustained attention processing (Li et al., 2012b). Consistent evidence is amassing for a specific role for the orbitofrontal gyrus in the pathophysiology of ADHD.

The ADHD cohort also showed significantly reduced nodal efficiency in right superior occipital gyrus; contrasting with significantly increased nodal efficiency in the left cuneus. Cuneus is a wedge-shaped area in the medial occipital lobe. In addition to its basic role in visual processing, gray matter volume in the cuneus has been suggested to be associated with better inhibitory control in bipolar depression patients (Haldane et al., 2008).

As mentioned in Introduction, resting-state fMRI, which study the properties of putative distributed brain networks assessing ongoing hemodynamic fluctuations while participants are in the resting-state in the scanner, has also been widely used in neuroimaging studies in ADHD. Using GTT, one such study reported significantly increased local efficiency of the Default Mode Network (DMN), and significantly decreased nodal efficiency in the medial prefrontal, temporal, and occipital regions in children with ADHD (Wang et al., 2009). On the other hand, a more recent resting-state fMRI study, by running both GTT and NBS in 90 cortical and subcortical regions, found no significantly altered global topological features, but abnormal inter-regional connectivity of the frontal-amgydala-occipital network and frontal-temporal-occipital network in young adults with ADHD (Cocchi, 2012). Most recently, Fair and colleagues analyzed resting-state data aggregated from a number of institutions worldwide, allowing for the assessment of resting-state network topologies in a very large cohort of ADHD children and early adolescents (*N* = 193) relative to an even larger control dataset (*N* = 455) (Fair et al., 2012). This study showed differences in a fronto-parietal and cerebellar systems, consistent with the notion that inattentiveness might arise because of deficits in task-control systems.

There are limitations to this study that need to be acknowledged. First, sex-related heterogeneity is likely to exist in ADHD (Rubia et al., 2009). To control for this possibility, we treated sex as a fixed-effect covariate during our analyses. Additional analyses, by comparing network efficiencies and hub profiles between the 12 boys and 10 girls within the patient group, showed no differences detected between the sexes in the patient group. Clearly, our test for potential sex-related differences is considerably underpowered, since the sample size was small. Second, head movement is always a significant concern in studies of network properties, and while we have very carefully controlled for it in the current study, and no significant differences between groups were seen, the development of improved methods for head-motion correction will undoubtedly further improve our sensitivity to this issue in future studies. Third, we applied pair-wise Pearson correlation over the nodes to construct the functional brain network. However, other correlation methods, such as partial correlation or constraint sparse partial correlation can also be considered to reduce spurious connections (Friedman et al., 2008; Wee et al., 2013). In addition, while a FDR correction has been applied, the analyses still involve a vast number of tests on a relatively small sample. Confidence in these complex findings would be greatly boosted by increasing the sample size, replication on another set or using some of the methods employed by network science to demonstrate the “robustness” of a network (such as examining the effect of “lesioning” or omitting certain nodes) in our future studies.

## Study limitations

As outlined in the methods section, controlling for head motion is a crucial issue in studies of functional connectivity, where relatively modest levels of motion can result in substantial image distortion and induce BOLD signal noise in both resting-state and task-based fMRI data. Further, more severe image distortion and signal noise will occur in voxels at the extremities of the brain volume when rotation along a perpendicular direction is incorporated. This likely explains prior results showing that long-range connections are more vulnerable in such calculations (Power et al., 2012). Using a very large sample size, Power and colleagues clearly demonstrated that head motion was associated with decreased long-range connectivity during resting-state scans. However, in a task-driven condition, the activation and connectivity patterns of the brain voxels are different from those during a resting-state, and as such, the result of head motion (even the same pattern of head motion) can be different. Our study is a clinical application of fMRI in ADHD, and given the relatively small sample sizes, we are not able to conclude that head motion caused either decreased or increased estimations of long-range connection in the task-driven data—we simply do not have enough power for such a validation. However, in the current study, we did collect both task-based and resting-state data in each participant and we found that head motion (by all the traditional and frame-wise measures) was significantly lower in the task-based data than it was in the resting-state data, a finding that held for both the ADHD and the control group. One plausible explanation for this difference is that the greater the focus required to perform the attention task may have resulted in a lower inclination to move. However, it will fall to future work in larger samples, using more than a single task, to ascertain if this is a consistently observed phenomenon. Regardless, for the purposes of this study, head motion was found to be minimal and there was no difference between groups in what little motion was observed. Since, the current study involves comparison of brain networks between groups, what minimal effects head motion may have had on our network analyses are unlikely to have impacted these between-groups comparisons.

## Author contributions

Dr. Ariane Sroubek contributed to subject recruitment, clinical assessment, and MRI data management. Drs. Shugao Xia and Xiaobo Li contributed to data analyses and results generation. Drs. Shugao Xia, John J. Foxe, Craig Branch, and Xiaobo Li contributed to manuscript writing.

### Conflict of interest statement

The authors declare that the research was conducted in the absence of any commercial or financial relationships that could be construed as a potential conflict of interest.
